# *Xanthomonas oryzae* pv. *oryzae* XopQ protein suppresses rice immune responses through interaction with two 14‐3‐3 proteins but its phospho‐null mutant induces rice immune responses and interacts with another 14‐3‐3 protein

**DOI:** 10.1111/mpp.12807

**Published:** 2019-05-15

**Authors:** Sohini Deb, Mahesh K. Gupta, Hitendra K. Patel, Ramesh V. Sonti

**Affiliations:** ^1^ CSIR‐Centre for Cellular and Molecular Biology (CSIR‐CCMB) Hyderabad 500007 India; ^2^ National Institute of Plant Genome Research New Delhi 110067 India; ^3^ Present address: Metahelix Life Sciences Ltd. Bangalore 560099 India

**Keywords:** *Xanthomonas oryzae* pv. *oryzae*, 14‐3‐3 protein, XopQ, resistance, rice, effector

## Abstract

Many bacterial phytopathogens employ effectors secreted through the type‐III secretion system to suppress plant innate immune responses. The *Xanthomonas* type‐III secreted non‐TAL effector protein Xanthomonas outer protein Q (XopQ) exhibits homology to nucleoside hydrolases. Previous work indicated that mutations which affect the biochemical activity of XopQ fail to affect its ability to suppress rice innate immune responses, suggesting that the effector might be acting through some other pathway or mechanism. In this study, we show that XopQ interacts in yeast and *in planta* with two rice 14‐3‐3 proteins, Gf14f and Gf14g. A serine to alanine mutation (S65A) of a 14‐3‐3 interaction motif in XopQ abolishes the ability of XopQ to interact with the two 14‐3‐3 proteins and to suppress innate immunity. Surprisingly, the S65A mutant gains the ability to interact with a third 14‐3‐3 protein that is a negative regulator of innate immunity. The XopQS65A mutant is an inducer of rice immune responses and this property is dominant over the wild‐type function of XopQ. Taken together, these results suggest that XopQ targets the rice 14‐3‐3 mediated immune response pathway and that its differential phosphorylation might enable interaction with alternative 14‐3‐3 proteins.

## Introduction

Plants can perceive pathogens by recognition of conserved molecular signatures of microorganisms, which are called as pathogen‐associated molecular patterns (or PAMPs, e.g. lipopolysaccharide or LPS) (Buttner and Bonas, 2010; Keshavarzi *et al.*, 2004) or the products released on cell/tissue damage, which are known as damage‐associated molecular patterns (DAMPs) (Bergey and Ryan, 1999; Jha *et al.*, 2007; Smith, 2001). Initial perception of pathogens involves recognition by receptors at the cell surface (Zipfel, 2008) and a subsequent signal cascade via protein kinase complexes (Lin *et al.*, 2014; Lu *et al.*, 2010). This results in activation of the expression of defence genes, leading to elaboration of defence responses (Felix *et al.*, 1999; Zipfel, 2008; Zipfel and Rathjen, 2008). These responses, which help plants counter a broad range of pathogens, are referred to as PAMP‐triggered immunity (PTI).

In order to counteract plant defence responses, many Gram‐negative phytopathogenic bacteria use effectors secreted through the type‐III secretion system (T3SS) to suppress PTI, helping in the establishment of the bacterium in the plant (Akimoto‐Tomiyama *et al.*, 2012; Hauck *et al.*, 2003; Jones and Dangl, 2006). Plants have in turn evolved resistance or ‘R’ genes which recognize either the effectors or the consequence of the action of effectors to trigger another layer of the plant immune responses that is referred to as effector‐triggered immunity (ETI). An additional layer is added to plant–pathogen interactions by the observation that some effectors secreted through the bacterial T3SS are able to suppress ETI.

How do effectors suppress plant innate immunity? These effectors have domains with functions similar to enzymes like phosphatases, kinases and acetyltransferases (Grant *et al.*, 2006; Kay and Bonas, 2009; Mudgett, 2005). Previous studies highlighted examples of pathways which are hijacked by type‐III effectors, e.g. the mitogen‐activated protein kinase (MAPK) pathway by XopAU (Teper *et al.*, 2018) or the proteasomal pathway by the interaction of the type‐III effector XopP with the E3 ubiquitin ligase PUB44 (Ishikawa *et al.*, 2014).

The 14‐3‐3 proteins are a class of eukaryotic proteins that play important roles in signal transduction cascades by binding to phosphor‐serine/threonine‐containing motifs in target proteins (Cotelle and Leonhardt, 2015). An increasing amount of evidence indicates that 14‐3‐3 proteins play important roles in regulating PTI and ETI (Lozano‐Duran and Robatzek, 2015). The 14‐3‐3 protein binding motif is conserved in a large number of bacterial effectors (Giska *et al.*, 2013) and they have been shown to interact with plant 14‐3‐3 proteins. The *Xanthomonas campestris* pv. *vesicatoria* (Xcv) XopN effector modulates plant defence responses by interaction with 14‐3‐3 scaffold proteins (Taylor *et al.*, 2012). The type‐III effector from *Xanthomonas campestris* pv. *vesicatoria* XopQ as well as its *Pseudomonas syringae* pv. *phaseolicola* homolog HopQ1 have the 14‐3‐3 protein‐binding motif (Giska *et al.*, 2013). HopQ1 interacts with the tomato 14‐3‐3 proteins TFT1 and TFT5, apparently in a phosphorylation‐dependent manner (Dubrow *et al.*, 2018; Li *et al.*, 2013). XopQ from Xcv has also been shown to function in the suppression of ETI by interaction with the tomato 14‐3‐3 protein TFT4 (Teper *et al.*, 2014).

*Xanthomonas oryzae* pv. *oryzae* is a Gram‐negative bacterium that causes bacterial blight, a serious disease of rice. Initial screening of a number of T3SS secreted effectors of *X. oryzae* pv. *oryzae* identified four secreted proteins, Xanthomonas outer protein N (XopN), XopQ, XopX and XopZ, as suppressors of cell wall damage induced innate immune responses in rice (Sinha *et al.*, 2013). Among these four proteins, only XopQ protein had a predicted biochemical activity. XopQ is highly conserved in Xanthomonads (Hajri *et al.*, 2009; Jalan *et al.*, 2013; Moreira *et al.*, 2010; Potnis *et al.*, 2011). Mutations in the XopQ protein that reduce biochemical activity did not seem to affect the ability of the protein to suppress rice innate immunity (Gupta *et al.*, 2015). The other structural feature of the XopQ protein is the presence of the 14‐3‐3 protein binding motif (Dubrow *et al.*, 2018; Giska *et al.*, 2013). Analysis of the amino acid sequence of *X. oryzae* pv. *oryzae* XopQ indicated the presence of two putative 14‐3‐3 binding motifs at amino acid residues 62‐67 (RTQSLP) and 219‐224 (RLATSP). In this study, we explored the role of the 14‐3‐3 protein binding motifs of XopQ in the suppression of rice innate immune responses. The results suggest that *X. oryzae* pv. *oryzae* XopQ can interact with several rice 14‐3‐3 proteins and that this interaction is important for the ability of the protein to modulate rice innate immunity.

## Results

### The *X. oryzae* pv*. oryzae* XopQ protein interacts with two rice 14‐3‐3 proteins

**Figure 1:**
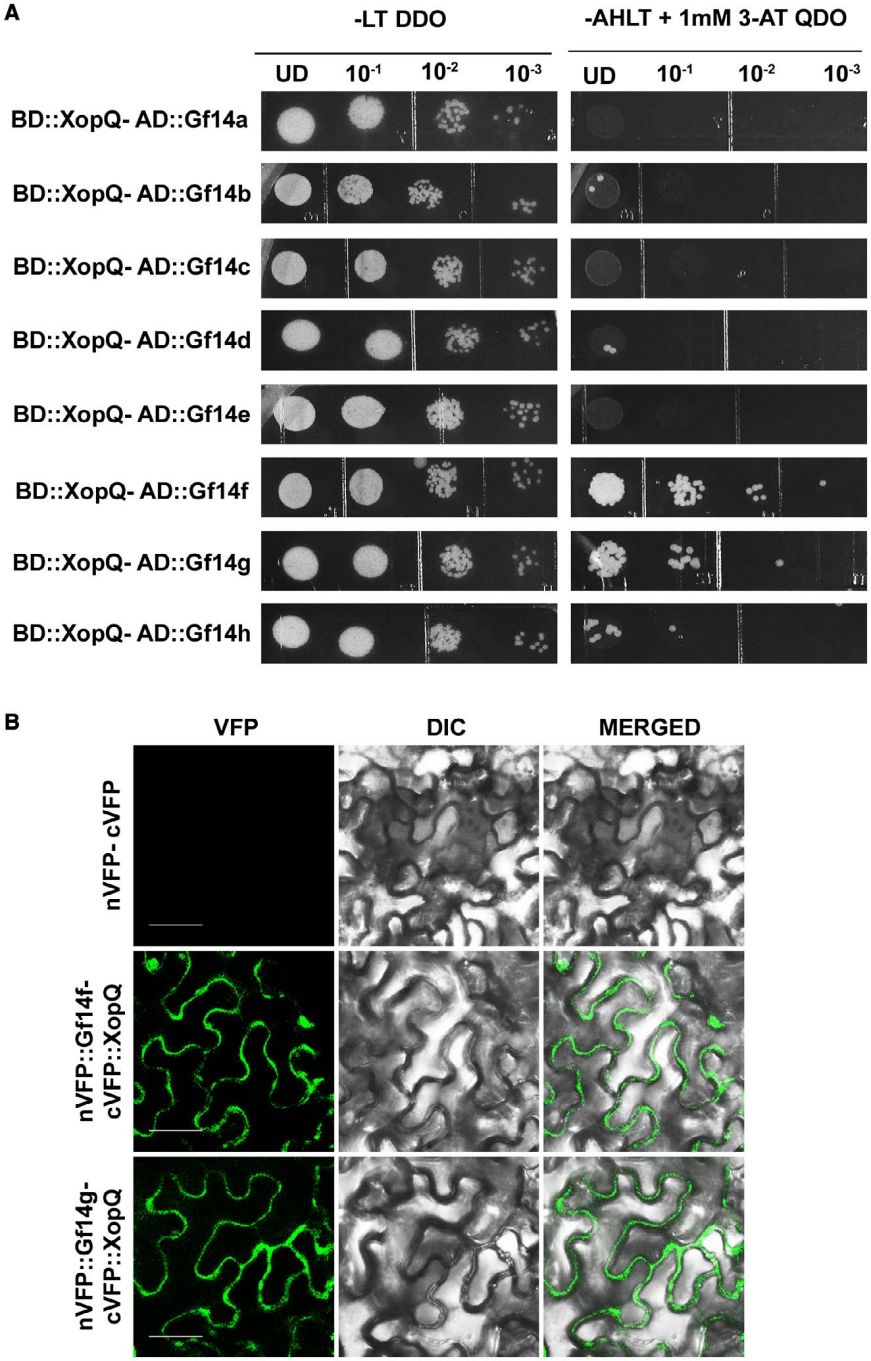
The *X. oryzae* pv. *oryzae* XopQ protein interacts with two rice 14‐3‐3 proteins, Gf14f and Gf14g. (A) Yeast strain pJ694a containing bait vector BD::XopQ was independently transformed with the following prey constructs: AD::Gf14a‐h. Transformed colonies were serially diluted and spotted on the non‐selective (SD‐LT; double dropout, DDO) and selective (SD‐ALTH; quadruple dropout, QDO) media with 1 mM 3‐amino‐1,2,4‐triazole. Observations were noted after 3 days of incubation at 30 °C. (B) For BiFC analysis of XopQ–14‐3‐3 interactions, leaves of *N. benthamiana* were hand‐infiltrated with a suspension (8 × 10^8^ CFU/mL) of two *A. tumefaciens* AGL1 strains containing empty vectors alone or *cVFP::XopQ* and *nVFP::Gf14f* or *nVFP::Gf14g*. Fluorescence was visualized in a confocal microscope (Carl Zeiss LSM880, Oberkochen, Germany) at 20× magnification and excitation wavelength (488 nm) 48 h after infiltration. Bar, 50 μm. The experiment was repeated three times with similar results.

Rice has eight 14‐3‐3 isoforms, namely, Gf14a, Gf14b, Gf14c, Gf14d, Gf14e, Gf14f, Gf14g and Gf14h. Since XopQ has two putative 14‐3‐3 protein binding motifs, we used the GAL‐4 based yeast two‐hybrid assay to test whether the XopQ protein can interact with rice 14‐3‐3 proteins. The XopQ protein was tagged with the DNA‐binding domain (BD) of the pDEST32 vector (Invitrogen) as bait, creating the fusion protein BD::XopQ, and each of the eight rice 14‐3‐3 proteins were tagged with the activation domain (AD) of the pDEST22 vector (Invitrogen), creating the AD::14‐3‐3 fusion proteins as prey. Transformation in the yeast strain pJ694A (James *et al.*, 1996) and subsequent selection on growth medium lacking adenine and histidine (reporter auxotrophic markers), leucine and tryptophan (vector selection markers) and containing 1,2,4‐triazole (3‐AT, a competitive inhibitor of *HIS3* biosynthesis) showed a strong interaction of the XopQ protein with the 14‐3‐3 proteins Gf14f and Gf14g (Fig. 1A). Expression of the remaining six rice 14‐3‐3 proteins in yeast was confirmed by western blotting, indicating that absence of interaction is not due to lack of expression (Supplementary Fig. S1).

In order to confirm this interaction *in planta*, we performed a bimolecular fluorescence complementation (BiFC) assay, with binary BiFC‐Gateway destination vectors (Gehl *et al.*, 2009). For this, the coding sequence of *XopQ* was cloned as a fusion protein with the C‐terminus of the Venus fluorescent protein (VFP) and the 14‐3‐3 genes* Gf14f* and *Gf14g* were cloned as fusion proteins with the N‐terminus of the VFP protein to yield the *cVFP‐XopQ*, *nVFP‐Gf14g* and *nVFP‐Gf14f* clones, respectively (Supplementary Table S1). The *Agrobacterium* strain AGL1 containing the independent expression constructs were hand‐infiltrated into *Nicotiana benthamiana* leaves for ectopic expression. On visualisation at 48 h post infiltration (hpi), the fluorescent protein signal was detected in the cytoplasm of the *N. benthamiana* cells in the interaction of XopQ wild‐type (WT) with Gf14g and Gf14f (Fig. 1B), which indicated that, *in planta*, these two proteins interact with the XopQ protein.

### The serine‐65 containing 14‐3‐3 protein binding motif of XopQ is essential for its interaction with the rice 14‐3‐3 proteins Gf14f and Gf14g

**Figure 2:**
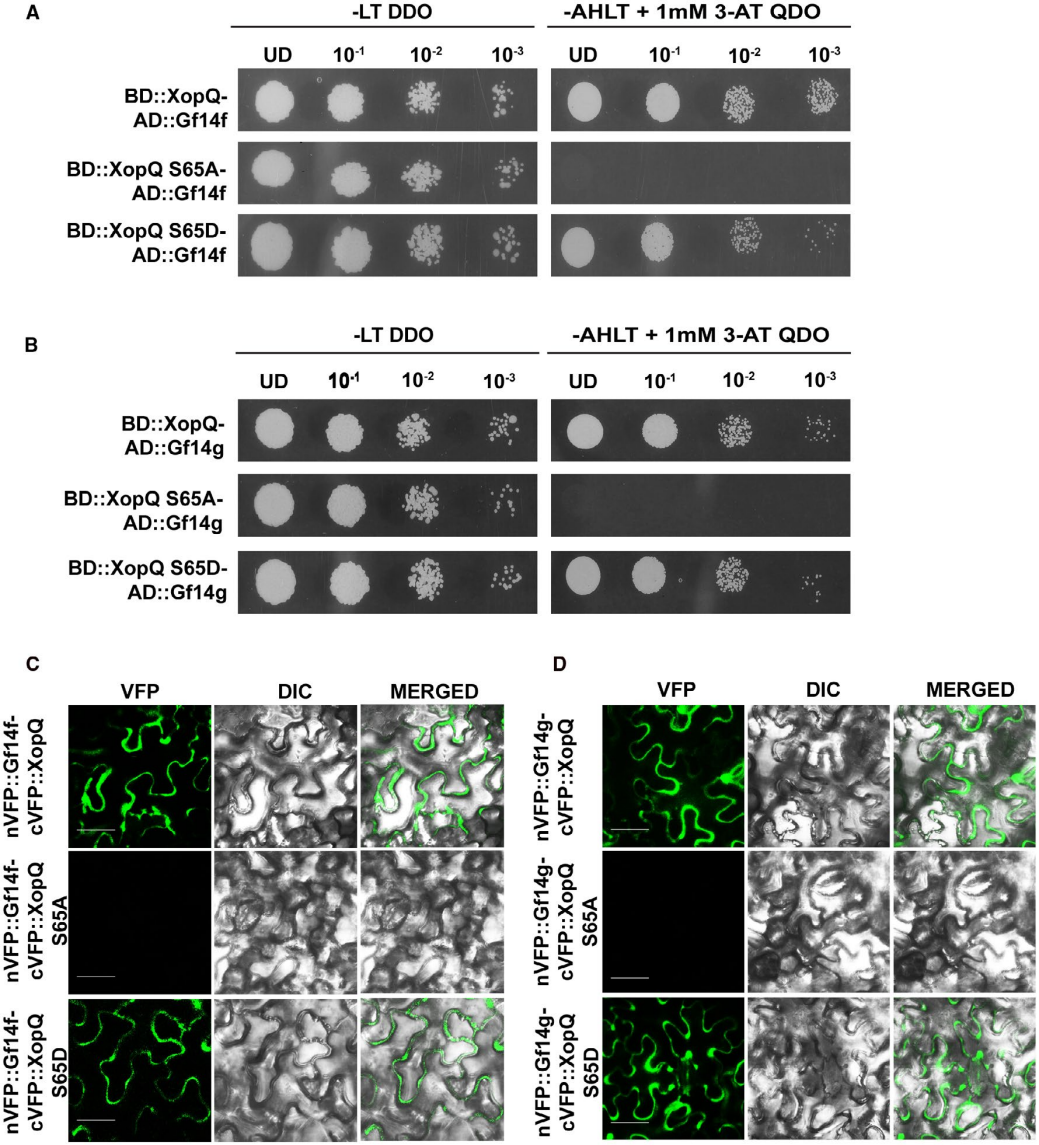
The serine‐65 containing motif‐1 14‐3‐3 protein binding motif of XopQ is essential for its interaction with the 14‐3‐3 proteins Gf14f and Gf14g. (A) Yeast two‐hybrid analysis. The yeast strain pJ694a carrying the bait vector pDEST32 containing *xopQ, xopQ S65A* or *xopQ S65D* was independently transformed with the following prey constructs: pDEST22 containing Gf14f or Gf14g. Transformed colonies were spotted on non‐selective (SD‐LT) and selective (SD‐ALTH) media with 1 mM 3‐amino‐1,2,4‐triazole and then incubated at 30 °C for 3 days. (B, C) BiFC analysis of XopQ–14‐3‐3 interactions in *N. benthamiana*. Leaves were hand‐infiltrated with a suspension (8 × 10^8^ CFU/mL total) of two *A. tumefaciens* strains containing *cVFP::xopQ*, *cVFP::xopQS65A* or *cVFP::xopQS65D* and *nVFP::Gf14f* or *nVFP::Gf14g*. Fluorescence was visualized in a confocal microscope (Zeiss LSM880) at 20× magnification and excitation wavelength (488 nm) 48 h after infiltration. Bar, 50 μm. Similar results were obtained in three independent experiments.

14‐3‐3 proteins are known to interact with the consensus 14‐3‐3 binding site in their client proteins in the event of phosphorylation of the conserved serine/threonine in the recognition motif. If Gf14f and Gf14g interact with XopQ via the 14‐3‐3 binding site, mutations that prevent phosphorylation of the motif should disrupt this interaction. Analysis of the amino acid sequence of *X. oryzae* pv. *oryzae* XopQ indicated the presence of two putative 14‐3‐3 binding motifs at amino acid residues 62 to 67 (RTQSLP) and 219 to 224 (RLATSP). Mutation of the conserved serine or threonine to alanine would render the motif phospho‐null whereas a mutation to aspartic acid would mimic the phosphorylated state of the serine/threonine residue and yield a phosphomimic mutant. Hence, first phospho‐null and phosphomimic mutants were made for the 14‐3‐3 protein binding motif encompassing serine‐65. The motif‐1 containing serine‐65 (*xopQS65A* and *xopQS65D*, respectively) was then cloned in the pDEST32 vector to create the BD‐ XopQ mutant fusion proteins. Furthermore, the ability of these proteins to interact with the 14‐3‐3 proteins Gf14f and Gf14g was assessed. In the yeast two‐hybrid assay, Gf14f (Fig. 2A) as well as Gf14g (Fig. 2B) lose interaction with the XopQ S65A protein, whereas they were seen to interact with XopQ S65D, indicating that phosphorylation of the serine‐65 residue at the 14‐3‐3 protein binding motif is important.

To study these interactions *in planta*, the coding sequences of the *XopQ* mutants (*xopQS65A* and *xopQS65D)* were cloned as fusion proteins with the C‐terminus of the Venus fluorescent protein (cVFP) to yield the *cVFP‐XopQS65A* and *cVFP‐XopQS65D* clones, respectively (Supplementary Table S1), and complementation of fluorescence was checked with the 14‐3‐3 proteins nVFP‐Gf14f and nVFP‐Gf14g. *In planta*, XopQS65A fails to interact with both Gf14f (Fig. 2C) and Gf14g (Fig. 2D), whereas cVFP::XopQS65D showed strong complementation of fluorescence with both nVFP::Gf14f (Fig. 2C) and nVFP::Gf14g (Fig. 2D). Stability of the XopQ protein was not affected by the serine‐65 to alanine mutation, as the XopQS65A protein could be detected in a western blot in rice (Supplementary Fig. S2).

### The serine‐65 containing 14‐3‐3 protein binding motif of XopQ is essential for suppression of rice immune responses

**Figure 3:**
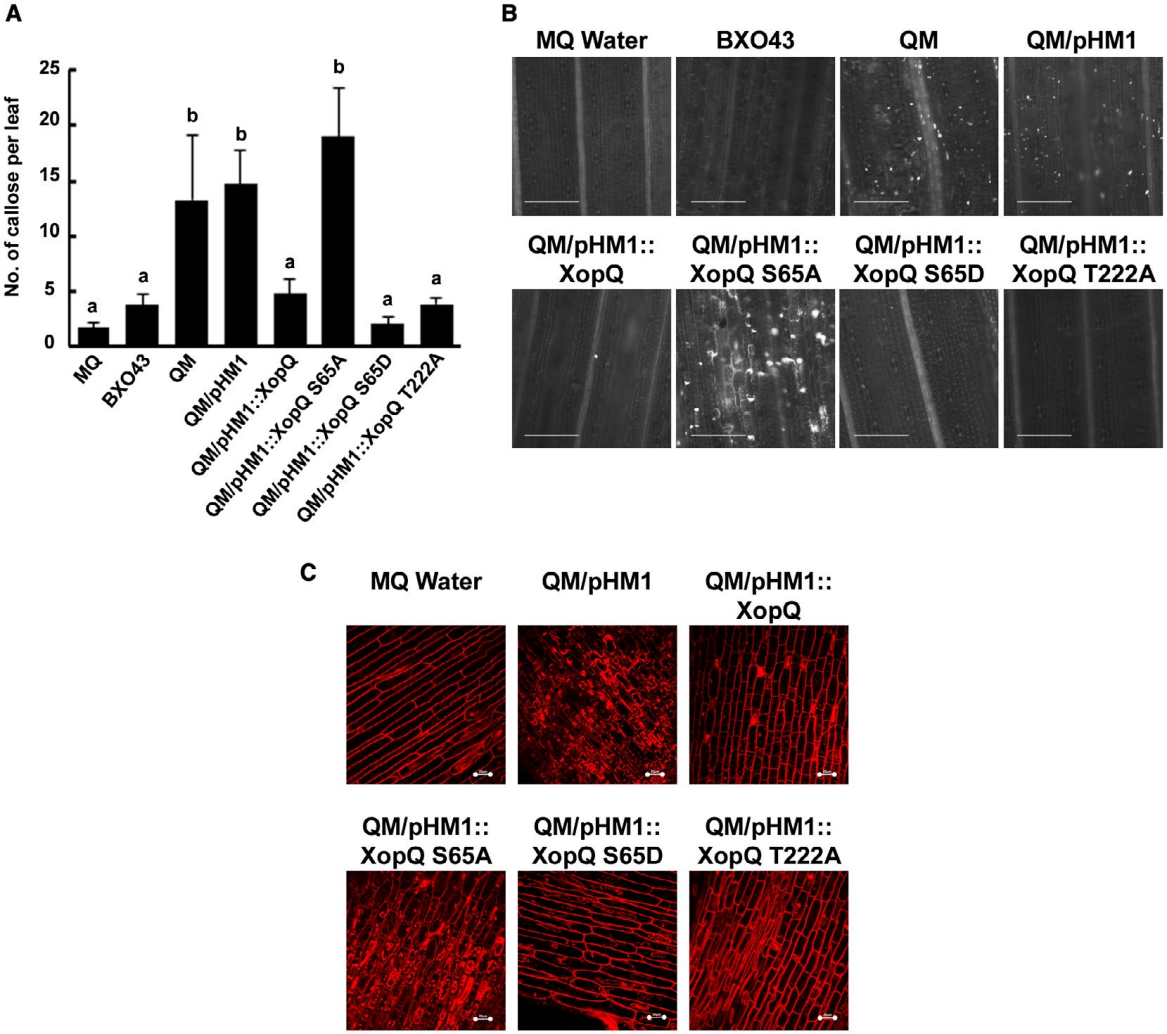
The serine‐65 containing 14‐3‐3 protein binding motif of XopQ is essential for suppression of rice immune responses. (A, B) For callose deposition assay, leaves of 2‐week‐old rice seedlings were infiltrated with one of the following: MilliQ water (MQ), *X. oryzae* pv. *oryzae* BXO43 (WT), *xopN xopQ xopX xopZ* QM, and QM harbouring the following plasmids: *pHM1, pHM1*::*xopQ*, *pHM1*::*xopQS65A*, *pHM1*::*xopQS65D* and* pHM1*::*xopQT222A*. The leaves were stained 16 h later with aniline blue and visualized under an epifluorescence microscope (365 nm). Mean and standard deviation were calculated for the number of callose deposits observed per leaf. Error bars indicate the standard deviation of readings from five infiltrated leaves. Columns in plots capped with the same letter were not significantly different from each other based on analysis of variance done using the Tukey–Kramer honestly significance difference test (*P < *0.05). Bar, 100 μm. The experiment was repeated three times and similar results were obtained. (C) Rice roots were treated with one of the following: water, *xopN xopQ xopX xopZ* QM containing the following constructs: *pHM1*, *pHM1*::*xopQ*, *pHM1*::*xopQS65A, pHM1*::*xopQS65D* or *pHM1*::*xopQT222A*. Treated roots were subsequently stained with PI and observed under a confocal microscope using 63× oil immersion objectives and an He‐Ne laser at 543 nm excitation to detect PI internalization. Five roots were imaged for each construct per experiment. Bar, 20 μm. Internalization of PI is indicative of defence response‐associated programmed cell death. Similar results were obtained in three independent experiments.

Previously our group has shown that in rice XopQ suppresses cell wall damage induced innate immune responses such as callose deposition and defence response associated programmed cell death (DRA‐PCD) (Sinha *et al.*, 2013). A positive reaction in the terminal deoxynucleotidyl transferase dUTP nick end labeling assay and inhibition by the baculoviral anti‐apoptotic protein p35 are hallmarks of PCD in rice (Hoang *et al.*, 2015). Similar results have been obtained by us for the DRA‐PCD seen in rice roots on treatment with cell wall degrading enzymes (unpublished observations). Hence, the effect of mutations in the 14‐3‐3 protein binding motif‐1 of XopQ (*xopQS65A* and *xopQS65D*) and motif‐2 (*xopQT222A*) in suppression of rice immune responses was assessed. A *xopN xopQ xopX xopZ* quadruple mutant (hereafter referred to as QM) is an inducer of host innate immune responses as it produces the elicitors of host innate immunity but is unable to suppress them (Sinha *et al.*, 2013). *XopQ* or its mutants were independently introduced into an *X. oryzae* pv. *oryzae* QM through the broad host range vector pHM1 and the resulting strains were assessed for their ability to suppress host immune responses. Introduction of the WT XopQ protein into the QM confers on it the ability to suppress callose deposition, a host innate immune response (Fig. 3A, B) (Gupta *et al.*, 2015). In contrast, the XopQS65A mutant is unable to suppress callose deposition when introduced into the QM strain (Fig. 3A, B). This indicates that the serine residue at the 65th position in the XopQ protein is important for the suppression of an immune response in rice. The phosphomimic mutant XopQS65D suppressed callose deposition as effectively as the XopQWT protein, indicating that phosphorylation of the serine‐65 residue is important for suppression of rice innate immune responses. The XopQT222A mutant suppresses callose deposition as well as the WT XopQ protein, indicating that this residue does not have a role in suppression of rice immune responses. Expression of the XopQ and XopQ mutant proteins *in planta* following *Xanthomonas* infection (Supplementary Fig. S2) was assessed by western blotting.

Treatment of rice roots with the QM induces plant DRA‐PCD. Treatment of rice roots with a QM strain carrying XopQ suppresses DRA‐PCD induced by the QM. However, treatment of rice roots with the QM strain carrying XopQS65A fails to suppress DRA‐PCD induced by QM (Fig. 3C). This indicates that mutation of the 14‐3‐3 binding motif of XopQ results in loss of the ability to suppress rice immune response associated PCD. Again, the XopQS65D phosphomimic mutant is able to suppress DRA‐PCD induced by QM. The XopQT222A mutant is as effective as the WT XopQ protein in suppressing DRA‐PCD induced by QM (Fig. 3C).

Overall, these results suggest that the serine‐65 containing 14‐3‐3 binding motif of XopQ but not the threonine‐222 containing 14‐3‐3 binding motif is required for suppression of rice innate immune responses and that phosphorylation of S65 appears to be required for this activity.

### Mutation of the serine‐65 containing motif‐1 14‐3‐3 protein binding motif of XopQ affects subcellular localization of the protein

**Figure 4:**
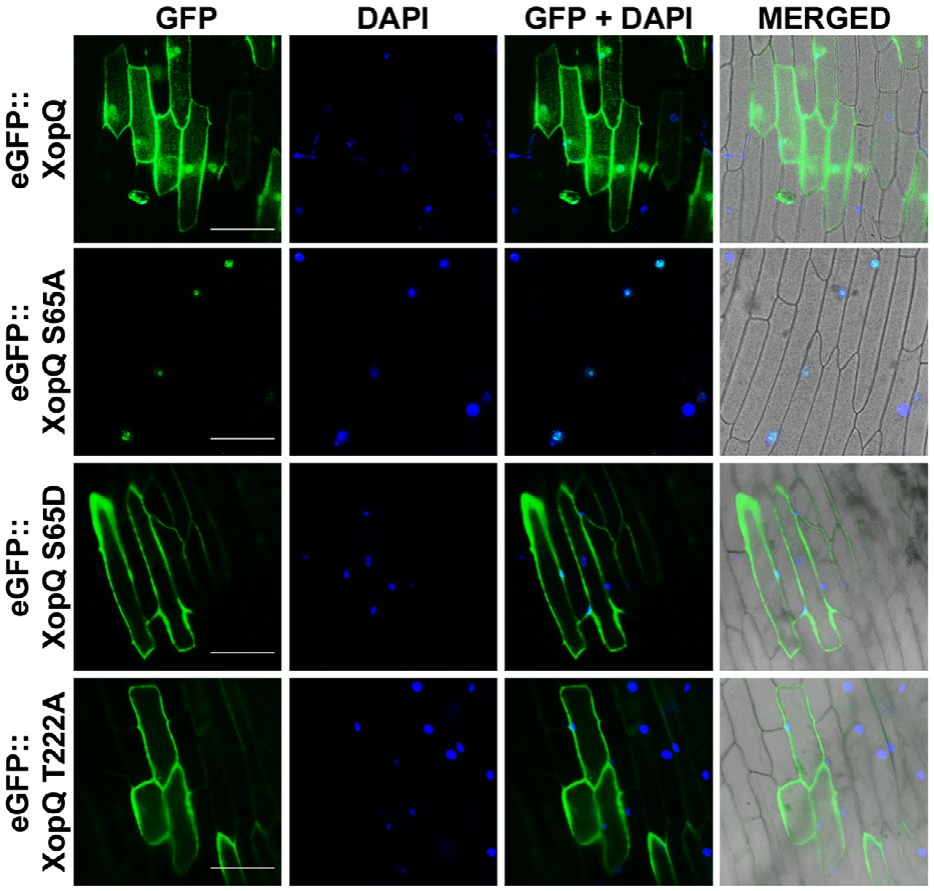
Mutations in the serine‐65 containing 14‐3‐3 protein binding motif of XopQ alters its subcellular localization. *Agrobacterium* strain AGL1 expressing one of the following was co‐cultivated with onion epidermal peels: *eGFP*::*xopQ*, *eGFP*::*xopQS65A*, *eGFP*::*xopQS65D* or *eGFP*::*xopQT222A*. Fluorescence was visualized in an epifluorescence microscope at 10× magnification and excitation wavelength (488 nm) 48 h after co‐cultivation. Bar, 100 μm. The experiment was repeated three times with similar results.

14‐3‐3 proteins are known to affect the biological function of their client proteins by alteration of their subcellular localization (Cotelle *et al.*, 2000; Paul *et al.*, 2012; Taoka *et al.*, 2011). Bioinformatic analysis of the XopQ sequence revealed a putative bipartite nuclear localization signal (NLS) using the NLS mapper tool (Kosugi *et al.*, 2009). Prediction of putative localization of XopQ was also done using the software TargetP, which gave an intermediate score (Nielsen *et al.*, 1997). The score indicated that this protein could putatively be localized to both the nucleus as well as the cytoplasm. In order to determine where *X. oryzae* pv. *oryzae* XopQ protein would localize, and whether mutation in the 14‐3‐3 protein binding motifs would change its localization, the *XopQWT*, *xopQS65A, xopQS65D* and *xopQT222A* mutants were cloned using the Gateway system into the pH7WGF2 binary vector containing the N‐terminal GFP tag (Karimi *et al.*, 2002) and transiently expressed in onion epidermal peels via agrobacterial delivery. Subcellular localization was checked by epifluorescence microscopy 48 h after co‐cultivation with *Agrobacterium*. DAPI staining was done to visualize the nucleus. The resulting eGFP::XopQWT fusion protein localized to both the nucleus and cytoplasm in onion epidermal cells (Fig. 4). This is in agreement with a previous report of GFP‐HopQ1 localization to the nucleus and cytoplasm (Kim *et al.*, 2009). However, the eGFP::XopQS65A mutant localized only in the nucleus as no signal could be seen in the cytoplasm (Fig. 4). On the other hand, the eGFP::XopQS65D mutant localizes mostly in the cytoplasm. The eGFP::XopQT222A also localized mostly in the cytoplasm. These results suggest that phosphorylation of S65 is required for localization of the XopQ protein in the cytoplasm.

### The XopQS65A mutant induces rice immune responses in a dominant manner over the WT protein

**Figure 5:**
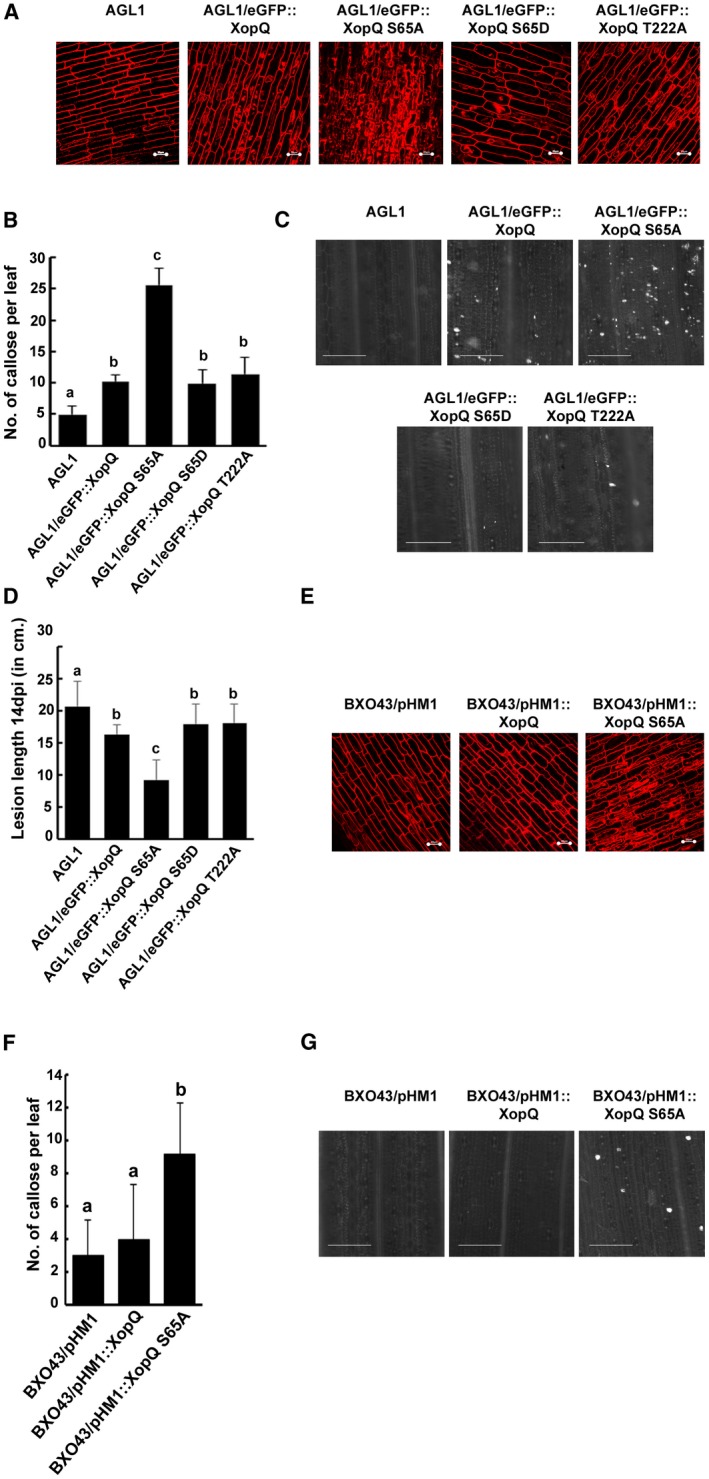
Overexpression of XopQ S65A induces rice innate immune responses in a dominant manner over WT XopQ function. (A) Rice roots (*n* = 5) were treated with *Agrobacterium* AGL1 alone or AGL1 containing one of the following: *eGFP*::*xopQ*, *eGFP*::*xopQS65A*, *eGFP*::*xopQS65D* or *eGFP*::*xopQT222A*. Treated roots were stained with PI and observed under a confocal microscope using 63× oil immersion objectives and an He‐Ne laser at 543 nm excitation to detect PI internalization. Bar, 20 μm. (B, C) For callose deposition assay, leaves of 14‐day‐old rice seedlings were infiltrated with one of the following: *Agrobacterium* AGL1 or AGL1 containing *eGFP*::*xopQ*, *eGFP*::*xopQ S65A*, *eGFP*::*xopQ S65D* or *eGFP*::*xopQ T222A*. The leaves were stained 16 h later with aniline blue and visualized under an epifluorescence microscope (365 nm). Mean and standard deviation were calculated for the number of callose deposits observed per leaf. Error bars indicate the standard deviation of readings from five inoculated leaves. Columns in plots capped with the same letter were not significantly different from each other based on analysis of variance done using the Tukey–Kramer honestly significance difference test (*P < *0.05). Bar, 100 μm. (D) The midveins of leaves of 40‐day‐old rice plants (susceptible variety TN‐1) (*n* = 5) were preinjected with *Agrobacterium* strain AGL1 alone or containing any one of the following: *eGFP*::*xopQ*, *eGFP*::*xopQ S65A*, *eGFP*::*xopQ S65D* or* eGFP*::*xopQ T222A*. Twelve hours later, the plants were infected with the WT *X. oryzae* pv. *oryzae* strain BXO43 using the pin‐prick inoculation method. The lesion lengths were measured 14 days post infection. Error bars indicate the standard deviation of readings from five inoculated leaves. Columns in plots capped with the same letter were not significantly different from each other based on analysis of variance done using the Tukey–Kramer honestly significance difference test (*P < *0.05). (E) Rice roots were treated with the WT *X. oryzae* pv. *oryzae* strain BXO43 containing one of the following: *pHM1*, *pHM1*::*xopQ* or *pHM1*::*xopQS65A*. Treated roots (*n* = 5) were stained with PI and observed under a confocal microscope using 63× oil immersion objectives and an He‐Ne laser at 543 nm excitation to detect PI internalization. Bar, 20 μm. (F, G) Leaves of 2‐week‐old rice seedlings were infiltrated with the WT *X. oryzae* pv. *oryzae* strain BXO43 containing one of the following: *pHM1*, *pHM1*::*xopQ* or *pHM1*::*xopQS65A*. The leaves were stained 16 h later with aniline blue and visualized under an epifluorescence microscope (365 nm). Mean and standard deviation were calculated for the number of callose deposits observed per leaf. Error bars indicate the standard deviation of readings from five inoculated leaves. Columns in plots capped with the same letter were not significantly different from each other based on analysis of variance done using the Tukey–Kramer honestly significance difference test (*P < *0.05). Bar, 100 μm.

Our observations suggested that the XopQS65A mutant showed high levels of immune markers (callose/DRA‐PCD) in the QM background. This could be due to its inability to suppress the QM‐induced immune responses, or due to the ability of the XopQS65A mutant itself to upregulate the immune responses. In order to test this possibility, 4‐day‐old rice seedlings were treated with *Agrobacterium* strain AGL1 containing the eGFP::XopQ or eGFP::XopQS65A fusion proteins and these roots were stained with propidium iodide (PI). Interestingly, treatment with the agrobacterial strain containing the eGFP::XopQS65A construct resulted in extensive internalization of PI as compared to eGFP::XopQ, suggesting that eGFP::XopQS65A is inducing a DRA‐PCD (Fig. 5A). Transient overexpression of XopQS65D or XopQT222A did not result in induction of DRA‐PCD. Similar results were obtained in a callose deposition assay wherein the *xopQS65A* mutant induces callose deposition significantly more than *xopQWT, xopQS65D* or *xopQT222A* (Fig. 5B and C). Also, prior treatment of rice leaves with agrobacterial strains carrying *xopQS65A*, but not those carrying *xopQ, xopQS65D* or *xopQT222A*, results in reduced lesion length on subsequent infection with *X. oryzae* pv. *oryzae* (Fig. 5D).

In order to assess if the XopQS65A mutation is dominant over WT XopQ, BXO43 strains having the empty vector pHM1 or *pHM1*::*xopQWT* or *pHM1*::*xopQS65A* were generated. Treatment of rice roots with the *BXO43/pHM1*::*xopQS65A* strain resulted in increased induction of DRA‐PCD as compared to treatment with BXO43 containing either empty vector *pHM1* or *pHM1*::*xopQWT* (Fig. 5E). Callose deposition induced by the *pHM1*::*xopQS65A* mutant was also higher as compared to either *pHM1* or *pHM1*::*xopQWT* (Fig. 5F,G). This suggests that the XopQS65A protein function is dominant over the WT XopQ function.

### XopQS65A mutant shows enhanced interaction with a novel 14‐3‐3 protein Gf14e

**Figure 6:**
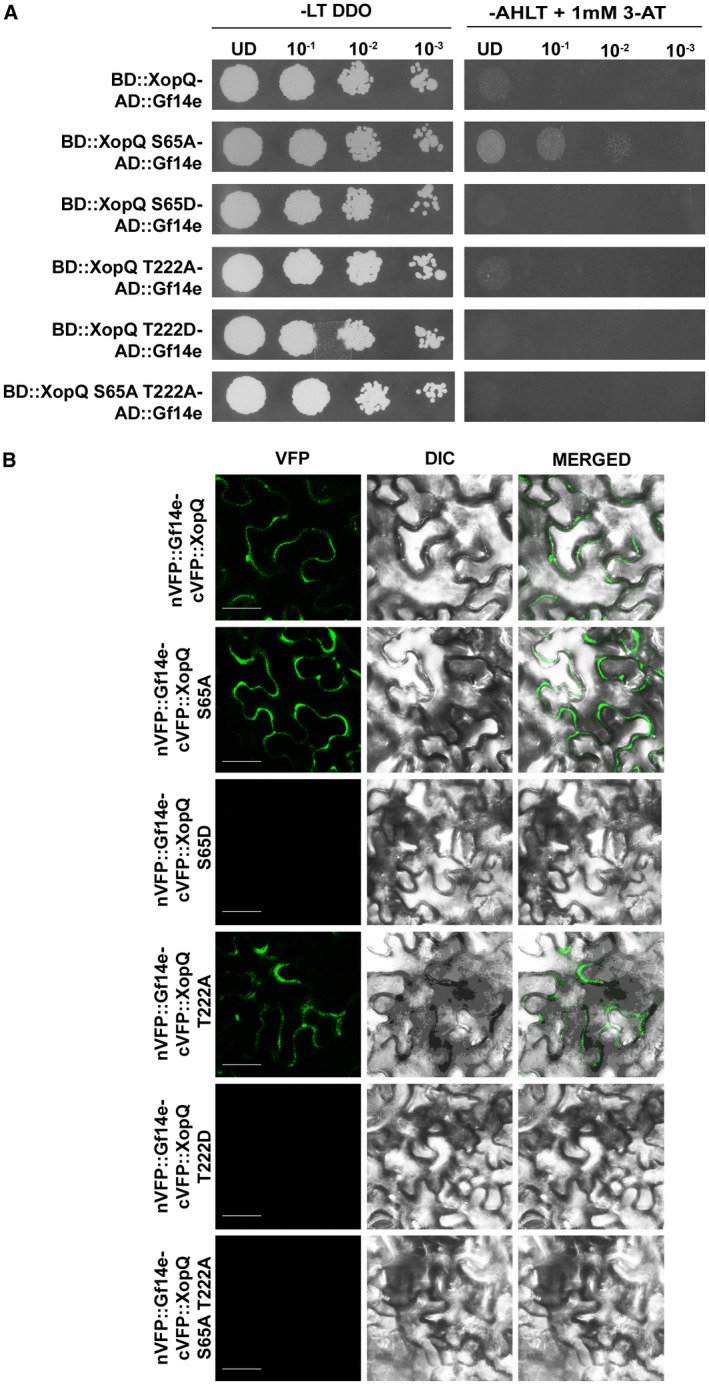
The XopQ S65A mutant interacts with a novel rice 14‐3‐3 protein, Gf14e. (A) Yeast two‐hybrid reporter strain pJ694a was co‐transformed with the pDEST32 vector containing *XopQ* or its 14‐3‐3 protein binding motif mutants *xopQ S65A*, *xopQ S65D*, *xopQ T222A*, *xopQ T222D* or *xopQ S65A T222A* and the pDEST22 vector containing 14‐3‐3 protein *Gf14e*, respectively. Transformants were selected on SD (−leu−trp) double dropout medium (DDO) and interaction was checked on SD (−leu−trp−his−ade) quadruple dropout medium (QDO) containing 1 mM 3‐amino‐1,2,4 triazole (3‐AT). (B) *Nicotiana benthamiana* leaves were co‐transformed with *Agrobacterium expressing* Gf14e:: N‐terminal of Venus Fluorescent protein (NE‐VFP) and XopQ:: C‐terminal of Venus Fluorescent protein (CE‐VFP), CE‐VFP::xopQS65A, CE‐VFP::xopQS65D, CE‐VFP::xopQT222A, CE‐VFP::xopQT222D or CE‐VFP ::xopQS65A‐T222A. Fluorescence was visualized in a confocal microscope (Carl Zeiss LSM880, Oberkochen, Germany) at 20× magnification and excitation wavelength (488 nm) 48 h after infiltration. Bar, 50 μm. Similar results were obtained in three independent experiments.

In order to explain how the XopQS65A mutant might be inducing rice innate immune responses, we hypothesised that the XopQS65A mutant might be interacting with a novel rice protein. For this, the ability of the XopQS65A mutant protein to interact with the eight rice 14‐3‐3 proteins was tested in a yeast two‐hybrid assay. Interestingly, in the yeast two‐hybrid assay, the XopQS65A mutant showed enhanced interaction with a different 14‐3‐3 protein, Gf14e, with which the XopQWT protein showed negligible interaction (Fig. 6A). XopQT222A showed a weak interaction with Gf14e similar to the interaction of WT XopQ with Gf14e (Fig. 6A). The XopQS65D and XopQT222D mutants failed to interact with the Gf14e protein, suggesting that constitutive phosphorylation at either one of the 14‐3‐3 protein‐binding motifs could be inhibiting interaction with this 14‐3‐3 protein. Also, the XopQS65A‐T222A mutant failed to interact with Gf14e, indicating that a functional 14‐3‐3 protein‐binding motif is required for this interaction. The results from the yeast two‐hybrid assay were also confirmed in an *in planta* BiFC assay (Fig. 6B).

## Discussion

The *X. oryzae* pv. *oryzae* type‐III effector XopQ is involved in suppression of rice immune responses. Since the biochemical activity of XopQ was shown not to be required for the suppression of rice immune responses, we hypothesized that it might be accomplishing this function by interaction with rice 14‐3‐3 proteins. XopQ has two 14‐3‐3 protein binding motifs, both of which are of the Mode‐I type, with Arg at position ‐3 (with respect to the phosphorylated residue) and proline at position + 2 (Muslin *et al.*, 1996; Yaffe *et al.*, 1997). In this study, we have studied the role of these 14‐3‐3 protein binding motifs of *X. oryzae* pv. *oryzae* XopQ in the modulation of rice immune responses. Our results show that abolishing the phosphorylation site by the serine‐65A mutation in the motif‐1 14‐3‐3 protein binding motifs of XopQ affects the following: (1) the ability of the protein to suppress rice immune responses, (2) the ability of the protein to interact with the rice 14‐3‐3 proteins Gf14f and Gf14g, and (3) the subcellular localization of the XopQ protein. A phosphomimic S65D mutant of XopQ is able to suppress rice immune responses, suggesting that phosphorylation of serine‐65 may be necessary for the ability of the XopQ protein to suppress host immunity. It is known that 14‐3‐3 proteins mostly interact with the consensus binding site in their client proteins when the motif is phosphorylated at the conserved serine/threonine (Cotelle and Leonhardt, 2015).

Earlier reports have highlighted the interaction of bacterial type‐III effectors with plant 14‐3‐3 proteins during the infection process. For example, interaction of *Xanthomonas campestris* pv. *vesicatoria* XopN with the tomato 14–3–3 TFT1 has been shown to be required for suppression of PTI (Taylor *et al.*, 2012). Also, the association of *P. syringae* HopQ1 with 14‐3‐3 proteins from tomato and *Nicotiana benthamiana* was shown to be phosphorylation dependent and was responsible for the modulation of HopQ1 subcellular localization and stability *in planta* (Giska *et al.*, 2013; Li *et al.*, 2013). Our results indicate that phosphorylation of the 14‐3‐3 protein binding motif affected the interaction of the XopQ protein with the host 14‐3‐3 proteins Gf14f and Gf14g, and its ability to suppress the rice innate immune responses as XopQS65A fails to interact with the cognate rice 14‐3‐3 proteins.

Suppression of ETI has been reported for the *Xcv* XopQ earlier (Teper *et al.*, 2014) wherein it suppresses immunity‐associated cell death induced by MAPK pathway genes and certain *R/avr* gene pairs. The 14‐3‐3 proteins are reported to play important roles in signal transduction during PTI as well as ETI. The presence of effectors that interact with 14‐3‐3 proteins may thus provide plant‐pathogenic bacteria with the ability to modulate PTI as well as ETI. Suppression of immune responses induced by the QM strain by the XopQ effector may be both suppression of ETI as well as suppression of DTI (damage‐triggered immunity) caused by the release of DAMPs by the QM strain.

Next, we asked whether mutations in the XopQ 14‐3‐3 protein binding motif would affect its subcellular localization. Earlier studies on the *Pseudomonas* effector protein HopQ1 indicated that it localizes in both the nucleus and the cytoplasm while an S51A mutation in the 14‐3‐3 binding site of this protein results in enrichment within the nucleus (Giska *et al.*, 2013). Our study also shows that the XopQS65A mutation results in a nuclear localization. This may be because of the inability of the XopQS65A mutant protein to interact with the 14‐3‐3 proteins Gf14f and Gf14g as earlier reports have suggested a role for 14‐3‐3 proteins in determining the subcellular localization of client proteins.

What might be the functional significance of the interaction of XopQ protein with Gf14f and Gf14g? It is possible that Gf14f and Gf14g might be involved in the signal transduction pathways that leads to elaboration of innate immunity and that XopQ is able to suppress innate immunity by interaction with these proteins. During this study, we also observed that transient expression of the XopQS65A mutant protein resulted in induction of defence responses such as callose deposition and DRA‐PCD in rice. Transient expression of XopQS65A protein also resulted in enhanced tolerance against subsequent *X. oryzae* pv. *oryzae* infection. Unlike the WT XopQ protein, the XopQS65A protein exhibits strong interaction with the rice 14‐3‐3 protein Gf14e. This protein has earlier been shown to be a negative regulator of rice innate immune responses as a knockdown line shows HR mimic lesions (Manosalva *et al.*, 2011). This raises the possibility that the ability of XopQS65A to induce immune rice responses is due to its ability to interact with Gf14e. Also, the inability of the XopQS65A‐T222A double mutant to interact with Gf14e suggests that the XopQS65A protein interacts with the latter protein via the 14‐3‐3 binding motif that encompasses T222. The inability of XopQS65D to interact with Gf14e suggests that phosphorylation at serine‐65 might be hindering interaction with Gf14e via threonine‐222 in the other motif. This might ensure that the binding of Gf14f and Gf14g at serine‐65 and of Gf14e do not occur at the same time. It is to be noted that the XopQT222A protein as well as XopQWT exhibit a minimal amount of interaction with Gf14e, suggesting that a basal level of interaction may be taking place via the serine‐65 containing motif. However, this level of interaction appears to be insufficient for the induction of rice immune responses.

What might be the significance in the observation that XopQ protein and XopQS65A interact with different 14‐3‐3 proteins? It is possible that the interaction of XopQS65A with Gf14e, a negative regulator of innate immunity, is an artifactual situation and that XopQ never interacts with this protein during infection. However, if XopQ can exist as a non‐phosphorylated form and interact with Gf14e, it can provide *X. oryzae* pv. *oryzae* with the ability to suppress as well as induce rice defence responses (including programmed cell death responses) during infection. This may be advantageous to *X. oryzae* pv. *oryzae* as it is considered to be a hemi‐biotroph which acts as a biotroph at early stages of infection and as a necrotroph at later stages of infection. The plant immune response pathways which the XopQ protein hijacks to accomplish its role during infection, its *in planta* phosphorylation status, and the rice proteins with which it might interact during infection remain to be explored.

## Experimental procedures

### Bacterial strains and plant material

The bacterial strains *Escherichia coli* DH5ɑ, *Agrobacterium tumefaciens* AGL1, *X. oryzae* pv. *oryzae* strain BXO43 (Thieme *et al.*, 2005) and *X. oryzae* pv. *oryzae* QM *X. oryzae* pv. *oryzae ΔxopQ ΔxopN ΔxopX ΔxopZ* (Sinha *et al.*, 2013) were used for the study. *E. coli* and *A. tumefaciens* were grown in Luria–Bertani (LB) medium. *E. coli* was grown at 37 °C whereas *A. tumefaciens* was grown at 28 °C. *X. oryzae* pv. *oryzae* strains were grown on peptone sucrose (PS) medium at 28 °C (Ray *et al.*, 2000). The yeast strain pJ694a was grown at 30 °C in yeast extract, peptone, dextrose (YPD) medium. The plant cultivars used were the susceptible rice variety Taichung Native‐1 (TN‐1) and *Nicotiana benthamiana*. The concentrations of antibiotics used were rifampicin (Rif) 50 μg/mL, spectinomycin (Sp) 50 μg/mL, gentamycin (Gent) 10 μg/mL, ampicillin (Amp) 100 μg/mL and kanamycin (Km) 15 μg/mL for *X. oryzae* pv. *oryzae* and 50 μg/mL for *E. coli*.

### Molecular biology and microbiology techniques

For the amplification and cloning of the WT copy of the *xopQ* gene and the respective 14‐3‐3 protein binding motif mutants as well as the rice 14‐3‐3 genes (Locus ID in Supplementary Table S2), high‐fidelity Phusion polymerase (Thermo Scientific, Waltham, Massachusetts, USA) was used. The genes were cloned into pENTR/D‐TOPO (Thermo Scientific, Waltham, Massachusetts, USA) and further by Gateway LR reaction (Thermo Scientific, Waltham, Massachusetts, USA) into Gateway compatible vectors. Taq polymerase from KAPA Biosystems (Wilmington, Massachusetts, USA) was used for all screening purposes. For cloning in the pHM1 vector, the primers as listed in Supplementary Table S3 were used for amplification of the *xopQ* gene or its 14‐3‐3 protein binding mutants using Phusion polymerase (Finnzymes). Restriction digestions were carried out using Thermo Fischer Scientific Fast Digest enzymes. Ligation reactions for cloning in pHM1 were carried out using T4 DNA ligase (Thermo Scientific, Waltham, Massachusetts, USA). Plasmids were purified using the alkaline lysis method. Gel extraction were carried out using a Macherey Nagel gel extraction kit. Agarose gel electrophoresis, transformation of *E. coli* and electroporation of plasmids into *X. oryzae* pv. *oryzae* were performed as described previously (Ray *et al.*, 2000; Subramoni and Sonti, 2005). All cloned vectors were confirmed by sequencing (ABI Prism 3700 automated DNA sequencer). The obtained sequences were subjected to homology searches using the BLAST algorithm in the National Center for Biotechnology Information database (Altschul *et al.*, 1990).

### Yeast two‐hybrid assays

The WT copy of *XopQ*, its respective 14‐3‐3 protein binding motif mutants and the eight rice 14‐3‐3 genes were cloned in the yeast two‐hybrid vectors pDEST32 and pDEST22 (Invitrogen) using the Gateway cloning system (Invitrogen). These plasmids were transformed into *Saccharomyces cerevisiae* strain pJ694a (James *et al.*, 1996). Yeast transformation was done as described (Gietz and Schiestl, 2007). Cells were plated on SD−LEU−TRP and grown at 30 °C to select for transformants. Colonies were scraped from plates, patched and grown overnight in SD−LEU−TRP liquid medium at 30 °C with shaking. The OD_600_ of saturated cultures was adjusted to 1.0 and serial dilutions were made and spotted on SD−LEU−TRP (selection for vector) and SD−LEU−TRP−ADE−HIS (selection for interaction) + 1 mM 3‐amino trizol (3‐AT; inhibitor of histidine biosynthesis) plates to identify rice 14‐3‐3 clones that interact with XopQ. The experiments were repeated three times.

### Yeast protein extraction

Protein extraction from yeast was done as described earlier (Taylor *et al.*, 2012). Briefly, yeast cells were resuspended in lysis buffer (1.85 M NaOH and 7% 2‐mercaptoethanol), lysed by vortexing with 0.5 mm glass beads and then proteins were precipitated in 10% trichloroacetic acid. Protein pellets after centrifugation were washed with 1 M Tris, pH 6.8 and then resuspended in Laemmli sample buffer. Equal amounts of isolated protein supernatants were further used for sodium dodecyl sulfate polyacrylamide gel electrophoresis (SDS‐PAGE) and western blotting.

### Bimolecular fluorescence complementation

The WT copy of x*opQ*, its respective 14‐3‐3 protein binding motif mutants and the eight rice 14‐3‐3 genes were cloned by Gateway cloning (Invitrogen) in the BIMOLECULAR FLUORESCENCE COMPLEMENTATION (BiFC) vectors pDEST‐VYNE(R)GW and pDEST‐VYCE(R)GW (Gehl *et al.*, 2009) carrying the N‐terminal and C‐terminal regions of the Venus fluorescent protein (VFP), respectively, to yield the constructs listed in Supplementary Table S1. The binary vectors obtained were then electroporated into the *Agrobacterium* strain AGL1. *Agrobacterium* cultures were grown to 0.8 OD_600_ and used for transient expression in *Nicotiana benthamiana*. VFP signals were examined 48 h after infiltration under a LSM880 confocal microscope (Carl Zeiss, Oberkochen, Germany) using 20× objectives and an He‐Ne laser at 488 nm excitation. Images were analysed using ZEN software. Five fields were imaged for each construct per experiment. Each set was repeated three times.

### Callose deposition in rice

Callose deposition assays were done as described earlier (Adam and Somerville, 1996; Hauck *et al.*, 2003; Sinha *et al.*, 2013; Tayi *et al.*, 2018). *X. oryzae* pv. *oryzae* strains were grown to saturation, OD_600_ adjusted to 1.0 using Milli‐Q water and infiltrated with a needleless 1 mL syringe into leaves of 14‐day‐old rice plants. 16 h after infiltration, the leaves were cut and placed in absolute alcohol at 65 °C to remove chlorophyll. This was followed by treatment with 70% ethanol at 65 °C and further by MQ water for rehydration. Subsequently, the samples were stained with 0.5% aniline blue solution prepared in 150 mM K_2_HPO_4_, pH 9.5. The leaves were then washed with MQ water and observed under an epifluorescence microscope (Nikon, Minato, Tokyo, Japan) using a blue filter (excitation wavelength of 365 nm) and 10× objective. Five leaves were imaged for each construct per experiment. Each set was repeated three times.

### Defence response associated programmed cell death assay

Assays for DRA‐PCD in rice roots were performed as described earlier (Sinha *et al.*, 2013; Tayi *et al.*, 2018). TN‐1 rice seeds were surface sterilized and germinated on 0.5% sterile agar for 3 days. Root tips 1 cm in length were cut from the seedlings and treated with *X. oryzae* pv. *oryzae* strains containing either *XopQWT* or its 14‐3‐3 binding mutants. After incubation for 16 h, roots were washed and stained with PI. The samples were visualized under a LSM‐510 Meta confocal microscope (Carl Zeiss) using 63× oil immersion objectives and an He‐Ne laser at 543 nm excitation to detect PI internalization. Images were analysed using LSM software. Five roots were imaged for each construct per experiment. Each set was repeated three times.

### *X. oryzae* pv. *oryzae* infection of rice

As described earlier (Pillai *et al.*, 2018), cultures of *Agrobacterium* strain AGL1 expressing an N‐terminal eGFP fusion to *XopQWT* or its 14‐3‐3 binding mutants were injected in the midveins of leaves of 40‐day‐old TN‐1 rice plants. At 12 h post injection, the leaves were infected with the WT *X. oryzae* pv. *oryzae* strain BXO43 by the pin‐prick method 1 cm above the point of injection. Progression of disease lesions was scored 14 days post infection. Five leaves were imaged for each construct per experiment. Each set was repeated three times.

### Western blotting of XopQ from exudate of rice leaves

Forty‐day‐old TN‐1 rice plants were clip‐inoculated with cultures of the *X. oryzae* pv. *oryzae* QM strain containing *XopQWT* or its respective 14‐3‐3 protein binding mutants. Twelve days after inoculation, the exudate from the infected leaves was collected and further used for sodium dodecyl sulfate polyacrylamide gel electrophoresis (SDS‐PAGE). XopQWT and its 14‐3‐3 binding mutants were detected by western blot analysis using anti:XopQ antibody (Gupta *et al.*, 2015). For immunoblotting using alkaline phosphatase (ALP), ALP conjugated to anti‐rabbit immunoglobulin G secondary antibody (Sigma; A3687‐1ML) was used. 4‐nitro blue tetrazolium (NBT, Roche‐11383213001) and 5‐bromo‐4‐chloro‐3‐indolyl‐phosphate, 4‐toluidine salt (BCIP, Roche‐11383221001) were used for detecting protein signals using the ALP buffer (100 mM NaCl, 100 mM Tris‐Cl pH 9.5, 50 mM MgCl_2_, 1% Tween‐20).

### Protein expression for localization in onion epidermal peels

Healthy onion scales (1 × 1 cm) were placed on a plate in such a way that their inner surfaces were immersed in *Agrobacterium* AGL1 containing *eGFP*::*XopQWT* or its 14‐3‐3 binding mutants (OD_600_ = 1–1.5) resuspended in a solution consisting of 5% (g/v) sucrose, 100 mg acetosyringone/L and 0.02% (v/v) Silwet‐77 for 12 h at 28 °C. Then the onion scales were transferred to plates of 1/2 MS (Murashige and Skoog salts, 30 g sucrose/L and 0.7% (g/v) agar, pH 5.7) and co‐cultivated with *Agrobacterium* for 2 days. Fluorescence was visualized under an epifluorescence microscope (Nikon) at 488 nm excitation and 10× objective. Each set was repeated three times.

## Conflict of interest

The authors declare that no competing interests exist.

## Supporting information


**Fig. S1** Expression of six rice 14‐3‐3 proteins in the yeast strain pJ694a following expression from yeast two‐hybrid vector pDEST22. Yeast two‐hybrid reporter strain pJ694a was transformed with the pDEST22 vector containing the respective rice 14‐3‐3 proteins. Transformants were selected on SD (−trp) dropout medium. Total cellular protein was isolated and checked for expression by western blotting. Expression of activation domain (AD) tagged proteins was detected by western blot using anti:AD antibody (Clontech GAL4 AD monoclonal antibody; 630402) raised in mouse. The secondary antibody of ALP conjugated to anti‐mouse IgG was used for detecting AD fusion protein expression (upper panel). Coomassie staining of the gel shows equal loading of protein in the different samples (lower panel).

**Fig. S2** Expression of *xopQ* gene of *Xanthomonas oryzae* pv. *oryzae* and its 14‐3‐3 protein‐binding motif mutants from exudate of rice leaves following *X. oryzae* pv. *oryzae* infection. Leaves of 40‐day‐old rice seedlings of Taichung Native 1 rice variety were clip inoculated with the following *X. oryzae* pv. *oryzae* strains: *XopQ‐,XopQ‐ /pHM1, XopQ‐/pHM1::XopQ, XopQ‐/pHM1::XopQ S65A, XopQ‐/pHM1::XopQ S65D and XopQ‐/pHM1::XopQ T222A*. Twelve days after inoculation, 3 cm leaf pieces from the inoculated end were cut and exudate was allowed to ooze out for 6 h at 4 °C. Expression of XopQWT and mutant proteins was detected by western blot analysis using anti:XopQ antibodies raised in rabbit. For immunoblotting using alkaline phosphatase (ALP), ALP conjugated to anti‐rabbit immunoglobulin G (Sigma, St. Louis, Missouri, USA; A3687 1ML) secondary antibody was used. XopQ expression was detected at 50 kDa (upper panel). Expression of the type II secretion system secreted enzyme lipase A was assessed by western blotting to normalize for protein loading by using anti‐lipase A antibody raised in rabbit (lower panel) and ALP based secondary antibody.

**Table S1** List of bacterial strains and plasmids used in this study (DOC).

**Table S2** Locus ID of the eight rice 14‐3‐3 genes (DOC).

**Table S3** List of oligonucleotide primers used in this study (DOC).

## Data Availability

The authors declare that the raw data will be available on request by mail to the corresponding author.
