# Supplementary figures and images for: Xanthomonas oryzae pv. oryzae XopQ protein suppresses rice immune responses through interaction with two 14‐3‐3 proteins but its phospho‐null mutant induces rice immune responses and interacts with another 14‐3‐3 protein
Source: Mol Plant Pathol. 2019 May 15;20(7):976–89. doi: 10.1111/mpp.12807 (PMC6856769; doi:10.1111/mpp.12807)

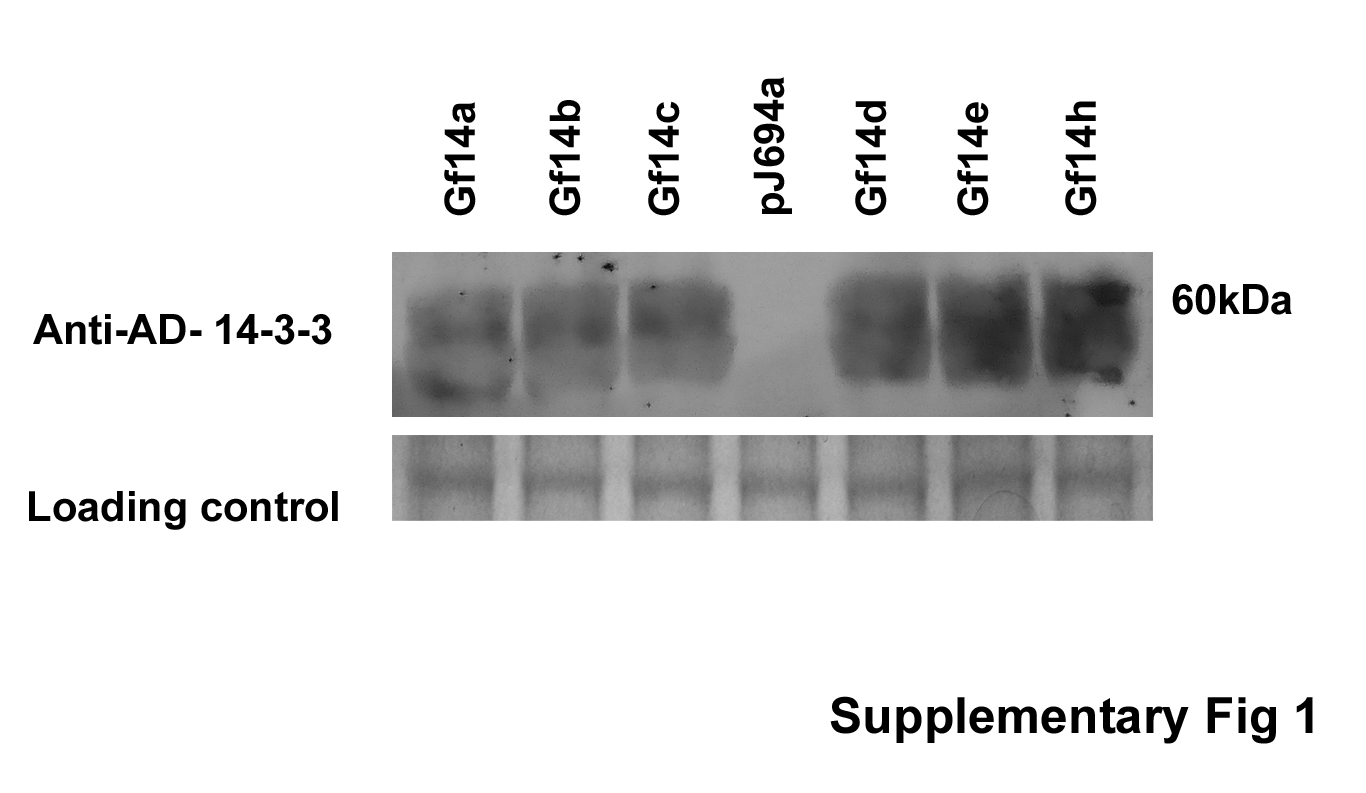

Supplement: Supplementary file 1 — Fig. S1 Expression of six rice 14‐3‐3 proteins in the yeast strain pJ694a following expression from yeast two‐hybrid vector pDEST22. Yeast two‐hybrid reporter strain pJ694a was transformed with the pDEST22 vector containing the respective rice 14‐3‐3 proteins. Transformants were selected on SD (−trp) dropout medium. Total cellular protein was isolated and checked for expression by western blotting. Expression of activation domain (AD) tagged proteins was detected by western blot using anti:AD antibody (Clontech GAL4 AD monoclonal antibody; 630402) raised in mouse. The secondary antibody of ALP conjugated to anti‐mouse IgG was used for detecting AD fusion protein expression (upper panel). Coomassie staining of the gel shows equal loading of protein in the different samples (lower panel). [file MPP-20-976-s001.tif]

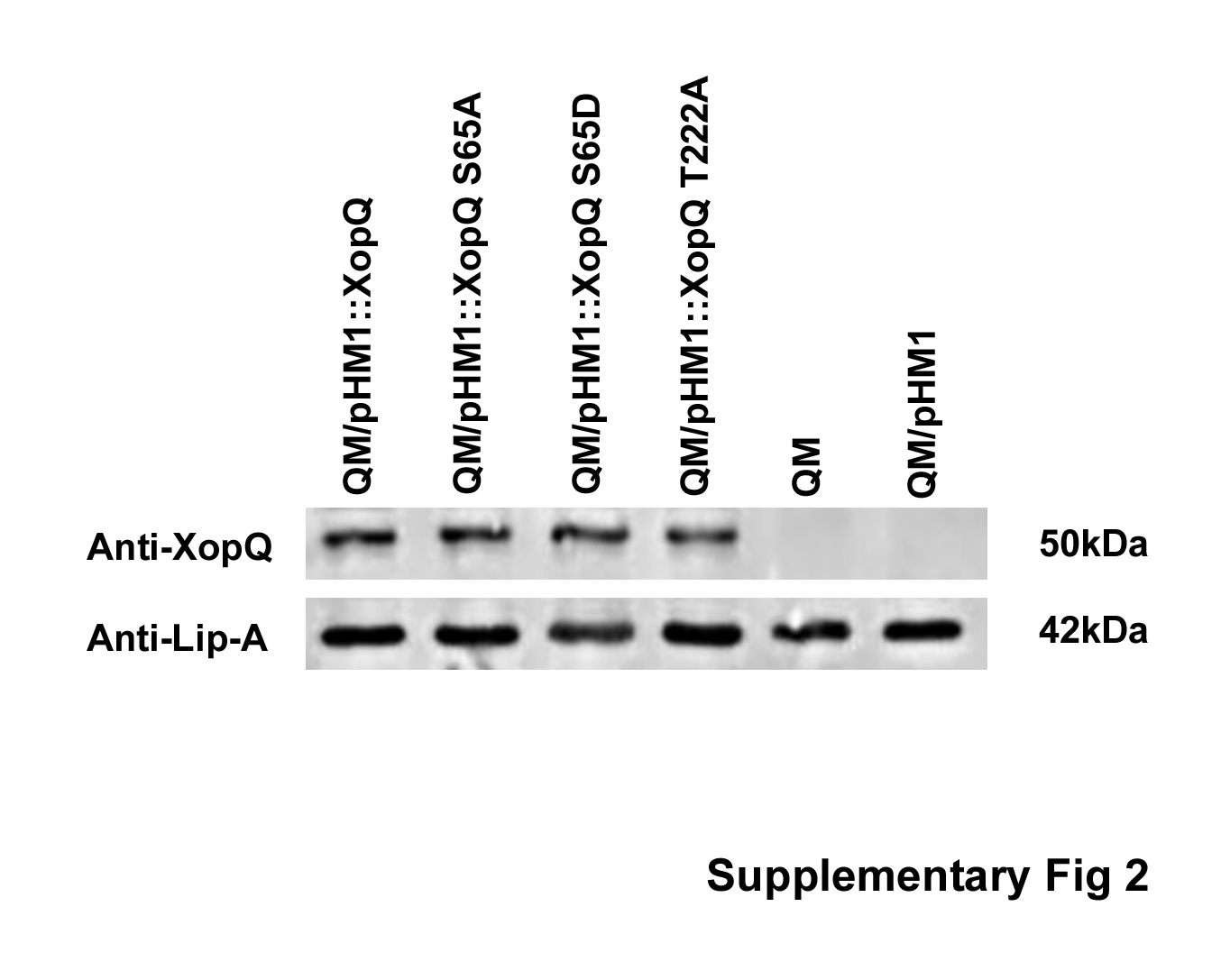

Supplement: Supplementary file 2 — Fig. S2 Expression of xopQ gene of Xanthomonas oryzae pv. oryzae and its 14‐3‐3 protein‐binding motif mutants from exudate of rice leaves following X. oryzae pv. oryzae infection. Leaves of 40‐day‐old rice seedlings of Taichung Native 1 rice variety were clip inoculated with the following X. oryzae pv. oryzae strains: XopQ‐,XopQ‐ /pHM1, XopQ‐/pHM1::XopQ, XopQ‐/pHM1::XopQ S65A, XopQ‐/pHM1::XopQ S65D and XopQ‐/pHM1::XopQ T222A. Twelve days after inoculation, 3 cm leaf pieces from the inoculated end were cut and exudate was allowed to ooze out for 6 h at 4 °C. Expression of XopQWT and mutant proteins was detected by western blot analysis using anti:XopQ antibodies raised in rabbit. For immunoblotting using alkaline phosphatase (ALP), ALP conjugated to anti‐rabbit immunoglobulin G (Sigma, St. Louis, Missouri, USA; A3687 1ML) secondary antibody was used. XopQ expression was detected at 50 kDa (upper panel). Expression of the type II secretion system secreted enzyme lipase A was assessed by western blotting to normalize for protein loading by using anti‐lipase A antibody raised in rabbit (lower panel) and ALP based secondary antibody. [file MPP-20-976-s002.tif]
