# Supplementary material for: Xanthomonas oryzae pv. oryzae XopQ protein suppresses rice immune responses through interaction with two 14‐3‐3 proteins but its phospho‐null mutant induces rice immune responses and interacts with another 14‐3‐3 protein
Source: Mol Plant Pathol. 2019 May 15;20(7):976–89. doi: 10.1111/mpp.12807 (PMC6856769; doi:10.1111/mpp.12807)
Supplement: Supplementary file 3 — Table S1 List of bacterial strains and plasmids used in this study (DOC). [file MPP-20-976-s003.docx]

**Supplementary Table S1. List of bacterial strains and plasmids used in this study**

| **Strains/Plasmids** | **Relevant Characteristics** | **Reference/Source** |
| --- | --- | --- |
| ***Escherichia coli* strains** |  |  |
| DH5α | λ^–^ f80dlacZDM15 D(lacZYA-argF) U169 recA1 endA hsdR17 (rK^–^ mK^–^) | Invitrogen |
|  |  |  |
| ***Xanthomonas oryzae* pv. *oryzae* strains** |  |  |
| BXO43 | *rif*-2; derivative of BXO1 (Wild type; Indian isolate) | Laboratory collection |
| *xopN xopQ xopX xopZ* | *xopN::*pK*18mob ΔxopQ ΔxopX ΔxopZ; rif-2;* derivative of BXO43; Km^r^ | Sinha *et al.*, 2013 |
| *xopN xopQ xopX xopZ/*pHM1 | *xopN::*pK*18mob ΔxopQ ΔxopX ΔxopZ/* pHM1*; rif-2;* Km^r^ ; Sp^r^  ; derivative of *xopN xopQ xopX xopZ* quadruple mutant | Sinha *et al.*, 2013 |
| *xopN xopQ xopX xopZ/*pHM1:: *xopQ* | *xopN::*pK*18mob ΔxopQ ΔxopX ΔxopZ/*pHM1:: *xopQ; rif-2;* Km^r^ ; Sp^r^  ; XopQ+, derivative of *xopN xopQ xopX xop*Z quadruple mutant | Gupta *et al.*, 2014 |
| *xopN xopQ xopX xopZ/*pHM1:: *xopQ S65A* | *xopN::*pK*18mob ΔxopQ ΔxopX ΔxopZ/*pHM1:: *xopQ S65A; rif-2;* Km^r^ ; Sp^r^  ; XopQS65A, derivative of *xopN xopQ xopX xopZ* quadruple mutant | This work |
| *xopN xopQ xopX xopZ/*pHM1:: *xopQ S65D* | *xopN::*pK*18mob ΔxopQ ΔxopX ΔxopZ/*pHM1:: *xopQ S65D; rif-2;* Km^r^ ; Sp^r^  ; XopQS65D, derivative of *xopN xopQ xopX xopZ* quadruple mutant | This work |
| *xopN xopQ xopX xopZ/*pHM1:: *xopQ T222A* | *xopN::*pK*18mob ΔxopQ ΔxopX ΔxopZ/*pHM1:: *xopQ T222A; rif-2;* Km^r^ ; Sp^r^  ; XopQT222A, derivative of *xopN xopQ xopX xopZ* quadruple mutant | This work |
|  |  |  |
| ***A. tumefaciens* strains** |  |  |
| AGL1 | AGL0 (*C58 pTiBo542*) *recA*::*bla*, T-region deleted Mop(+) Cb(R) (AGL0 is an EHA101 with the T-region deleted) | Lazo *et al.*, 1991 |
| AGL1/ pH7WGF2 | AGL1 carrying plasmid pH7WGF2; Sp^r^ | This work |
| AGL1/ eGFP::*xopQ* | AGL1 carrying plasmid pH7WGF2::*xopQ*; Sp^r^ | This work |
| AGL1/ eGFP::*xopQ S65A* | AGL1 carrying plasmid pH7WGF2::*xopQ S65A*; Sp^r^ | This work |
| AGL1/ eGFP::*xopQ S65D* | AGL1 carrying plasmid pH7WGF2::*xopQ S65D*; Sp^r^ | This work |
| AGL1/ eGFP::*xopQ T222A* | AGL1 carrying plasmid pH7WGF2::*xopQ T222A*; Sp^r^ | This work |
| AGL1/pDEST-VYCE(R)GW | AGL1 carrying plasmid pDEST-VYCE(R)GW; Km^r^ | This work |
| AGL1/pDEST-VYNE(R)GW | AGL1 carrying plasmid pDEST-VYNE(R)GW; Km^r^ | This work |
| AGL1/cVFP::*xopQ* | AGL1 carrying plasmid pDEST-VYCE(R)GW::*xopQ,*  Km^r^ | This work |
| AGL1/cVFP::*xopQ S65A* | AGL1 carrying plasmid pDEST-VYCE(R)GW::*xopQ S65A*; Km^r^ | This work |
| AGL1/cVFP::*xopQ S65D* | AGL1 carrying plasmid pDEST-VYCE(R)GW::*xopQ S65D*; Km^r^ | This work |
| AGL1/cVFP::*xopQ T222A* | AGL1 carrying plasmid pDEST-VYCE(R)GW::*xopQ T222A*; Km^r^ | This work |
| AGL1/cVFP::*xopQ T222D* | AGL1 carrying plasmid pDEST-VYCE(R)GW::*xopQ T222D*; Km^r^ | This work |
| AGL1/cVFP::*xopQ S65A T222A* | AGL1 carrying plasmid pDEST-VYCE(R)GW::*xopQ S65A T222A*; Km^r^ | This work |
| AGL1/nVFP::*gf14e* | AGL1 carrying plasmid pDEST-VYNE(R)GW::*gf14e*; Km^r^ | This work |
| AGL1/nVFP::*gf14f* | AGL1 carrying plasmid pDEST-VYNE(R)GW::*gf14f*; Km^r^ | This work |
| AGL1/nVFP::*gf14g* | AGL1 carrying plasmid pDEST-VYNE(R)GW::*gf14g*; Km^r^ | This work |
|  |  |  |
| ***Saccharomyces cerevisiae* strains** |  |  |
| pJ694a | MATa trp1-901 leu2-3,112 ura3-52 his3-200 gal4(deleted) gal80(deleted) LYS2::GAL1-HIS3 GAL2-ADE2 met2::GAL7-lacZ | James P. *et al.*, 1996 |
| pJ694a/pDEST32 | pJ694a carrying plasmid pDEST32 ; -LEU | This work |
| pJ694a/pDEST22 | pJ694a carrying plasmid pDEST22; -TRP | This work |
| pJ694a/BD::*xopQ* | pJ694a carrying plasmid pDEST32::*xopQ;* -LEU | This work |
| pJ694a/BD::*xopQ S65A* | pJ694a carrying plasmid pDEST32::*xopQ S65A*; -LEU | This work |
| pJ694a/BD::*xopQ S65D* | pJ694a carrying plasmid pDEST32::*xopQ S65D;* -LEU | This work |
| pJ694a/BD::*xopQ T222A* | pJ694a carrying plasmid pDEST32::*xopQ T222A*; -LEU | This work |
| pJ694a/BD::*xopQ T222D* | pJ694a carrying plasmid pDEST32::*xopQ T222D;* -LEU | This work |
| pJ694a/BD::*xopQ S65A T222A* | pJ694a carrying plasmid pDEST32::*xopQ S65A T222A;* -LEU | This work |
| pJ694a/AD::*gf14a* | pJ694a carrying plasmid pDEST22::*gf14a; -*TRP | This work |
| pJ694a/AD::*gf14b* | pJ694a carrying plasmid pDEST22::*gf14b;* -TRP | This work |
| pJ694a/AD::*gf14c* | pJ694a carrying plasmid pDEST22::*gf14c; -*TRP | This work |
| pJ694a/AD::*gf14d* | pJ694a carrying plasmid pDEST22::*gf14d; -*TRP | This work |
| pJ694a/AD::*gf14e* | pJ694a carrying plasmid pDEST22::*gf14e; -*TRP | This work |
| pJ694a/AD::*gf14f* | pJ694a carrying plasmid pDEST22::*gf14f; -*TRP | This work |
| pJ694a/AD::*gf14g* | pJ694a carrying plasmid pDEST22::*gf14g; -*TRP | This work |
| pJ694a/AD::*gf14h* | pJ694a carrying plasmid pDEST22::*gf14h; -*TRP | This work |
|  |  |  |
| **Plasmids** |  |  |
| pENTR/D-TOPO | cloning vector; Km^r^ | Invitrogen |
| pENTR/D-TOPO::*xopQ* | pENTR/D-TOPO with 1,392-bp fragment containing full length *xopQ* gene from BXO43 genomic DNA; Km^r^ | This work |
| pENTR/D-TOPO::*xopQ S65A* | pENTR/D-TOPO with 1,392-bp fragment containing full length *xopQ S65A* gene from BXO43; Km^r^ | This work |
| pENTR/D-TOPO::*xopQ S65D* | pENTR/D-TOPO with 1,392-bp fragment containing full length *xopQ S65D* gene from BXO43; Km^r^ | This work |
| pENTR/D-TOPO::*xopQ T222A* | pENTR/D-TOPO with 1,392-bp fragment containing full length *xopQ T222A* gene from BXO43; Km^r^ | This work |
| pENTR/D-TOPO::*xopQ T222D* | pENTR/D-TOPO with 1,392-bp fragment containing full length *xopQ T222D* gene from BXO43; Km^r^ | This work |
| pENTR/D-TOPO::*xopQ S65A T222A* | pENTR/D-TOPO with 1,392-bp fragment containing full length *xopQS65A T222A* gene from BXO43; Km^r^ | This work |
| pENTR/D-TOPO::*gf14a* | pENTR/D-TOPO with 795-bp fragment containing full length *gf14a* gene from cDNA of Cellulase A(Sigma) treated rice leaves; Km^r^ | This work |
| pENTR/D-TOPO::*gf14b* | pENTR/D-TOPO with 789-bp fragment containing full length *gf14b* gene from cDNA of Cellulase A(Sigma) treated rice leaves; Km^r^ | This work |
| pENTR/D-TOPO::*gf14c* | pENTR/D-TOPO with 771-bp fragment containing full length *gf14c* gene from cDNA of Cellulase A(Sigma) treated rice leaves; Km^r^ | This work |
| pENTR/D-TOPO::*gf14d* | pENTR/D-TOPO with 798-bp fragment containing full length *gf14d* gene from cDNA of Cellulase A(Sigma) treated rice leaves; Km^r^ | This work |
| pENTR/D-TOPO::*gf14e* | pENTR/D-TOPO with 789-bp fragment containing full length *gf14e* gene from cDNA of Cellulase A(Sigma) treated rice leaves; Km^r^ | This work |
| pENTR/D-TOPO::*gf14f* | pENTR/D-TOPO with 783-bp fragment containing full length *gf14f* gene from cDNA of Cellulase A(Sigma) treated rice leaves; Km^r^ | This work |
| pENTR/D-TOPO::*gf14g* | pENTR/D-TOPO with 612-bp fragment containing full length *gf14g* gene from cDNA of Cellulase A(Sigma) treated rice leaves; Km^r^ | This work |
| pENTR/D-TOPO::*gf14h* | pENTR/D-TOPO with 693-bp fragment containing full length *gf14h* gene from cDNA of Cellulase A(Sigma) treated rice leaves; Km^r^ | This work |
| pMG2 (pHM1::*xopQ*) | pHM1 with 1,392-bp fragment containing full length *xopQ* gene from BXO43 cloned into HindIII and KpnI sites of pHM1; Sp^r^ | Gupta *et al.*, 2014 |
| pSoD1 | pHM1 with 1,392-bp fragment containing full length *xopQ S65A* gene from BXO43 cloned into HindIII and KpnI sites of pHM1; Sp^r^ | This work |
| pSoD2 | pHM1 with 1,392-bp fragment containing full length *xopQ S65D* gene from BXO43 cloned into HindIII and KpnI sites of pHM1; Sp^r^ | This work |
| pSoD3 | pHM1 with 1,392-bp fragment containing full length *xopQ T222A* gene from BXO43 cloned into HindIII and KpnI sites of pHM1; Sp^r^ | This work |
| pH7WGF2 | Plant overexpression vector with constitutive 35S CaMV promoter and N-terminal GFP fusion; Sp^r^ | Karimi *et al.*, 2000 |
| eGFP::*xopQ* | pH7WGF2 with 1,392-bp fragment containing full length *xopQ* gene obtained from recombination of pENTR::*xopQ* with pH7WGF2; Sp^r^ | This work |
| eGFP::*xopQ S65A* | pH7WGF2 with 1,392-bp fragment containing full length *xopQ S65A* gene obtained from recombination of pENTR::*xopQ S65A* with pH7WGF2; Sp^r^ | This work |
| eGFP::*xopQ S65D* | pH7WGF2 with 1,392-bp fragment containing full length *xopQ S65D* gene obtained from recombination of pENTR::*xopQ S65D* with pH7WGF2; Sp^r^ | This work |
| eGFP::*xopQ T222A* | pH7WGF2 with 1,392-bp fragment containing full length *xopQ T222A* gene obtained from recombination of pENTR::*xopQ T222A* with pH7WGF2; Sp^r^ | This work |
| pDEST-VYCE(R)GW | Plant expression vector for BiFC having C-terminal portion of VFP as an N-terminal fusion to protein of interest; Km^r^ | Gehl *et* *al.*, 2009 |
| pDEST-VYNE(R)GW | Plant expression vector for BiFC having N-terminal portion of VFP as an N-terminal fusion to protein of interest; Km^r^ | Gehl *et* *al.*, 2009 |
| cVFP::*xopQ* | pDEST-VYCE(R)GW with 1,392-bp fragment containing full length *xopQ* gene obtained from recombination of pENTR::*xopQ* with pDEST-VYCE(R)GW; Km^r^ | This work |
| cVFP::*xopQ S65A* | pDEST-VYCE(R)GW with 1,392-bp fragment containing full length *xopQ S65A* gene obtained from recombination of pENTR::*xopQ S65A*with pDEST-VYCE(R)GW; Km^r^ | This work |
| cVFP::*xopQ S65D* | pDEST-VYCE(R)GW with 1,392-bp fragment containing full length *xopQ S65D* gene obtained from recombination of pENTR::*xopQ S65D* with pDEST-VYCE(R)GW; Km^r^ | This work |
| cVFP::*xopQ T222A* | pDEST-VYCE(R)GW with 1,392-bp fragment containing full length *xopQ T222A* gene obtained from recombination of pENTR::*xopQ T222A* with pDEST-VYCE(R)GW; Km^r^ | This work |
| cVFP::*xopQ T222D* | pDEST-VYCE(R)GW with 1,392-bp fragment containing full length *xopQ T222D* gene obtained from recombination of pENTR::*xopQ T222D* with pDEST-VYCE(R)GW; Km^r^ | This work |
| cVFP::*xopQ* *S65A T222A* | pDEST-VYCE(R)GW with 1,392-bp fragment containing full length *xopQ S65A T222A* gene obtained from recombination of pENTR::*xopQ S65A T222A*with pDEST-VYCE(R)GW; Km^r^ | This work |
| nVFP::*gf14e* | pDEST-VYNE(R)GW with 789-bp fragment containing full length *gf14e* gene obtained from recombination of pENTR::*gf14e* with pDEST-VYNE(R)GW; Km^r^ | This work |
| nVFP::*gf14f* | pDEST-VYNE(R)GW with 783-bp fragment containing full length *gf14f* gene obtained from recombination of pENTR::*gf14f* with pDEST-VYNE(R)GW; Km^r^ | This work |
| nVFP::*gf14g* | pDEST-VYNE(R)GW with 612-bp fragment containing full length *gf14g* gene obtained from recombination of pENTR::*gf14g* with pDEST-VYNE(R)GW; Km^r^ | This work |
| pDEST32 | Expression vector for yeast-2-hybrid having the binding domain (BD) of a transcription factor as an N-terminal fusion to the gene of interest for activation of reporter genes; Gent^r^ | Invitrogen |
| pDEST22 | Expression vector for yeast-2-hybrid having the activation domain (AD) of a transcription factor as an N-terminal fusion to the gene of interest for activation of reporter genes; Amp^r^ | Invitrogen |
| BD::*xopQ* | pDEST32 with 1,392-bp fragment containing full length *xopQ* gene obtained from recombination of pENTR::*xopQ* with pDEST32; Gent^r^ | This work |
| BD::*xopQ S65A* | pDEST32 with 1,392-bp fragment containing full length *xopQ S65A* gene obtained from recombination of pENTR::*xopQ S65A* with pDEST32; Gent^r^ | This work |
| BD::*xopQ S65D* | pDEST32 with 1,392-bp fragment containing full length *xopQ S65D* gene obtained from recombination of pENTR::*xopQ S65D* with pDEST32; Gent^r^ | This work |
| BD::*xopQ T222A* | pDEST32 with 1,392-bp fragment containing full length *xopQ T222A* gene obtained from recombination of pENTR::*xopQ T222A* with pDEST32; Gent^r^ | This work |
| BD::*xopQ T222D* | pDEST32 with 1,392-bp fragment containing full length *xopQ T222D* gene obtained from recombination of pENTR::*xopQ T222D* with pDEST32; Gent^r^ | This work |
| BD::*xopQ S65A T222A* | pDEST32 with 1,392-bp fragment containing full length *xopQ S65A T222A* gene obtained from recombination of pENTR::*xopQ S65A T222A* with pDEST32; Gent^r^ | This work |
| AD::*gf14a* | pDEST22 with 795-bp fragment containing full length *gf14a* gene obtained from recombination of pENTR::*gf14a* with pDEST22; Amp^r^ | This work |
| AD::*gf14b* | pDEST22 with 789-bp fragment containing full length *gf14b* gene obtained from recombination of pENTR::*gf14b* with pDEST22; Amp^r^ | This work |
| AD::*gf14c* | pDEST22 with 771-bp fragment containing full length *gf14c* gene obtained from recombination of pENTR::*gf14c* with pDEST22; Amp^r^ | This work |
| AD::*gf14d* | pDEST22 with 798-bp fragment containing full length *gf14d* gene obtained from recombination of pENTR::*gf14d* with pDEST22; Amp^r^ | This work |
| AD::*gf14e* | pDEST22 with 789-bp fragment containing full length *gf14e* gene obtained from recombination of pENTR::*gf14e* with pDEST22; Amp^r^ | This work |
| AD::*gf14f* | pDEST22 with 783-bp fragment containing full length *gf14f* gene obtained from recombination of pENTR::*gf14f* with pDEST22; Amp^r^ | This work |
| AD::*gf14g* | pDEST22 with 612-bp fragment containing full length *gf14g* gene obtained from recombination of pENTR::*gf14g* with pDEST22; Amp^r^ | This work |
| AD::*gf14h* | pDEST22 with 693-bp fragment containing full length *gf14h* gene obtained from recombination of pENTR::*gf14h* with pDEST22; Amp^r^ | This work |
