# Supplementary material for: Xanthomonas oryzae pv. oryzae XopQ protein suppresses rice immune responses through interaction with two 14‐3‐3 proteins but its phospho‐null mutant induces rice immune responses and interacts with another 14‐3‐3 protein
Source: Mol Plant Pathol. 2019 May 15;20(7):976–89. doi: 10.1111/mpp.12807 (PMC6856769; doi:10.1111/mpp.12807)
Supplement: Supplementary file 4 — Table S2 Locus ID of the eight rice 14‐3‐3 genes (DOC). [file MPP-20-976-s004.docx]

**Supplementary Table S2. Locus ID of rice 14-3-3 genes from MSU database.**

| **Rice 14-3-3s** | **MSU accession no.** | **CDS Length** | **Protein Size**  **(in kDa)** |
| --- | --- | --- | --- |
| *Gf14a* | LOC_Os08g37490 | 795 bp | 29 |
| *Gf14b* | LOC_Os04g38870 | 789 bp | 29.87 |
| *Gf14c* | LOC_Os08g33370 | 771 bp | 28.83 |
| *Gf14d* | LOC_Os11g34450 | 798 bp | 29.27 |
| *Gf14e* | LOC_Os02g36974 | 789 bp | 29.7 |
| *Gf14f* | LOC_Os03g50290 | 783 bp | 29.18 |
| *Gf14g* | LOC_Os01g11110 | 612 bp | 22.95 |
| *Gf14h* | LOC_Os11g39540 | 693 bp | 25.86 |
