# Supplementary material for: Xanthomonas oryzae pv. oryzae XopQ protein suppresses rice immune responses through interaction with two 14‐3‐3 proteins but its phospho‐null mutant induces rice immune responses and interacts with another 14‐3‐3 protein
Source: Mol Plant Pathol. 2019 May 15;20(7):976–89. doi: 10.1111/mpp.12807 (PMC6856769; doi:10.1111/mpp.12807)
Supplement: Supplementary file 5 — Table S3 List of oligonucleotide primers used in this study (DOC). [file MPP-20-976-s005.docx]

**Supplementary Table S3. List of primers used in the study.**

| **PRIMER LIST** | | | |
| --- | --- | --- | --- |
| **NAME** | **SEQUENCE** | **PURPOSE/ DETAILS** | **SOURCE** |
| M13F | GTAAAACGACGGCCAGT | Sequencing | Lab collection |
| M13R | GGAAACAGCTATGACCATG | Sequencing | Lab collection |
| XopQF | CACCATGCAGCCCACCGCAATCCG | Cloning in pENTR-D/TOPO; Screening | This work |
| XopQR | TCAGCGCGCATGTTCCCCCTCGT | Cloning in pENTR-D/TOPO; Screening | This work |
| Gf14aF | ATGGTGTACATGGCGAAGCTGGCG | Cloning in pENTR-D/TOPO; Screening | This work |
| Gf14aR | CTAGTGCTCATCCTCAGGCTTGGTTGC | Cloning in pENTR-D/TOPO; Screening | This work |
| Gf14bF | CACCATGTCGGCACAGGCGGAGCTTT | Cloning in pENTR-D/TOPO; Screening; Semi-quant RT-PCR | This work |
| Gf14bR | TTACTGCCCCTCGCTGGAGTCG | Cloning in pENTR-D/TOPO; Screening; Semi-quant RT-PCR | This work |
| Gf14cF | CACCATGTCTCGGGAGGAGAATGTCTACATGGC | Cloning in pENTR-D/TOPO; Screening; Semi-quant RT-PCR | This work |
| Gf14cR | TTACTGGCCCTCGCAGGCGTC | Cloning in pENTR-D/TOPO; Screening; Semi-quant RT-PCR | This work |
| Gf14dF | CACCATGTCGCCGGCGGAGCC | Cloning in pENTR-D/TOPO; Screening | This work |
| Gf14dR | TCACTGATCCCCAGGCTCTTTTGGAG | Cloning in pENTR-D/TOPO; Screening | This work |
| Gf14eF | CACCATGTCGCAGCCTGCTGAGCTTTCC | Cloning in pENTR-D/TOPO; Screening; Semi-quant RT-PCR | This work |
| Gf14eR | TCACTGTCCATCTCCTGATTCGCCCT | Cloning in pENTR-D/TOPO; Screening; Semi-quant RT-PCR | This work |
| Gf14fF | CACCATGTCGCCTGCTGAGGCATCG | Cloning in pENTR-D/TOPO; Screening | This work |
| Gf14fR | TTAGTGGCCCTCTCCTTCAGGCTTC | Cloning in pENTR-D/TOPO; Screening | This work |
| Gf14gF | CACCATGGCCCCCAGCGACGAC | Cloning in pENTR-D/TOPO; Screening | This work |
| Gf14gR | CTAGGTATAGATCAGAGCAGGCAAGGAATGAGAAACA | Cloning in pENTR-D/TOPO; Screening | This work |
| Gf14hF | CACCATGAAGGAGAGGGAGAAGGTGGTGC | Cloning in pENTR-D/TOPO; Screening | This work |
| Gf14hR | TTAGCCCTCCATAACAACATCATCGTCCTTAGATGT | Cloning in pENTR-D/TOPO; Screening | This work |
| XopQ-pHM1 F | TCCCAAGCTTATGCAGCCCACCGCAATCCG | Cloning in pHM1 vector/contains HindIII site | This work |
| XopQ-pHM1 R | TCGGGGTACCTCAGCGCGCATGTTCCCCCTCGT | Cloning in pHM1 vector/contains KpnI site | This work |
| XopQ S65A SDM F | CAGGCCTTGCCCGCGCGA | Site directed mutagenesis using XopQ as template | This work |
| XopQ S65A SDM R | CAAGGCCTGCGTGCGGCG | Site directed mutagenesis using XopQ as template | This work |
| XopQ S65D SDM F | CAGGACTTGCCCGCGCGA | Site directed mutagenesis using XopQ as template | This work |
| XopQ S65D SDM R | CAAGTCCTGCGTGCGGCG | Site directed mutagenesis using XopQ as template | This work |
| XopQ T222A SDM F | GCGGCCTCGCCCCACAAG | Site directed mutagenesis using XopQ as template | This work |
| XopQ T222A SDM R | CGAGGCCGCCAGGCGCTC | Site directed mutagenesis using XopQ as template | This work |
| XopQ T222D SDM F | GCGGACTCGCCCCACAAG | Site directed mutagenesis using XopQ as template | This work |
| XopQ T222D SDM R | CGAGTCCGCCAGGCGCTC | Site directed mutagenesis using XopQ as template | This work |
| eGFP F | ATGGTGAGCAAGGGCGAGGAG | Screening for pH7WGF2 vector clones | This work |
| eGFP R | TTACTTGTACAGCTCGTCCATGCCGAG | Screening for pH7WGF2 vector clones | This work |
